# Learning curve in pipeline embolization device: results from the pipeline embolization device in China post-market multicentre registry study

**DOI:** 10.1097/JS9.0000000000000467

**Published:** 2023-05-09

**Authors:** Junlin Lu, Yang Zhao, Hongqi Zhang, Tianxiao Li, Donglei Song, Sheng Guan, Aisha Maimaitili, Yunyan Wang, Wenfeng Feng, Yang Wang, Jieqing Wan, Guohua Mao, Huaizhang Shi, Xinjian Yang, Jianmin Liu, Yuanli Zhao

**Affiliations:** a Department of Neurosurgery, West China Hospital, Sichuan University, Sichuan; b Peking University International Hospital; c Beijing Tiantan Hospital; d Xuanwu Hospital, Capital Medical University, Beijing; e Zhengzhou University People’s Hospital; f First Affiliated Hospital of Zhengzhou University, Zhengzhou; g Shanghai Donglei Brain Hospital; h Renji Hospital, School of Medicine, Shanghai Jiao Tong University; i Changhai Hospital, Naval Medical University, Shanghai; j First Affiliated Hospital of Xinjiang Medical University, Uruqi; k Qilu Hospital of Shandong University, Jinan; l Nanfang Hospital, Southern Medical University, Guangzhou; m First Affiliated Hospital of Nanchang University; n Second Affiliated Hospital of Nanchang University, Nanchang; o First Affiliated Hospital of Harbin Medical University, Harbin, China.

**Keywords:** Cumulative summation analysis, Complications, Learning curve, Outcomes, Pipeline embolization device

## Abstract

**Background:**

Intracranial aneurysms pose a significant health issue, affecting 3–5% of the adult population. The pipeline embolization device (PED) has emerged as a promising treatment for these lesions. This study aimed to investigate the impact of operator experience on complication and poor outcome rates, as well as the learning curve for PED.

**Methods:**

A total of 217 patients were consecutively enroled from four eligible centres and divided into three groups based on the number of procedures performed: group 1 (first 10 procedures), group 2 (11–20 procedures), and group 3(>20 procedures). Major complications include operation-related ischaemic or haemorrhagic events and mass effect deterioration. Poor outcome was defined as a modified Rankin Scale score greater than 2 at discharge. Cumulative summation (CUSUM) analysis was generated to assess the learning curve according to major complications and poor outcome.

**Results:**

The study found that major complications and poor outcomes occurred in 5.1% and 2.3% of cases, respectively. The rate of major complications decreased from 10.0% in group 1 to 2.9% in group 3 (*P*=0.053), while the rate of poor outcomes decreased from 7.5% in group 1 to 0.7% in group 3 (*P*=0.015). Multivariable regression analysis adjusted for covariates showed that operator experience was associated with a lower rate of poor outcomes (*P*=0.034). CUSUM analysis demonstrated that the learning curve for avoiding major complications and poor outcomes required 27 (mean=13) and 40 (mean=20) cases, respectively.

**Conclusions:**

These findings suggest that PED treatment requires a learning curve of 40 cases to achieve reproducibility regarding complications and functional results. Additionally, major complications and poor outcomes significantly decreases after the first 20 procedures. CUSUM analysis can serve as a useful tool for monitoring and assessing surgical performance.

## Introduction

Highlights

- First to investigate the learning curve of pipeline embolization device treatment using cumulative summation analysis.
- The operator experience and major complications and poor outcomes were linear.
- Major complication and poor outcome rate significantly decreases after the first 20 procedures.

The prevalence of unruptured intracranial aneurysms in the general population is estimated to be 3–5%, with the incidence increasing with age^1^. With the development and increased use of neuroimaging, the detection of intracranial aneurysms has become more frequent^2^. Since the introduction of Guglielmi detachable coils in 1991, endovascular management options for intracranial aneurysms have continued to improve^3^. One of these options, the pipeline embolization device (PED), is a low porosity flow-diverting cylindrical device designed to treat intracranial aneurysms through endoluminal parent vessel reconstruction. Several large cohort studies have demonstrated the safety and efficacy of PED^4–7^, making it a standard first-line option for treating an increasing population of intracranial aneurysms at many centres. However, experience with the device in non-large neurovascular centres remains somewhat preliminary. The significance of training and retention of learning the PED technique cannot be overstated. Studies have shown that the acquisition of technical skills in surgical procedures is essential for reducing complications and improving patient outcomes. Additionally, the retention of these skills is vital for maintaining procedural safety and achieving long-term success^8^.

Despite the growing use of PED for intracranial aneurysm treatment, there remains an information gap regarding the learning curve and technical competency required for this procedure. Specifically, it is unclear how many procedures are necessary to achieve minimal technical competence and procedural safety, and what factors influence the learning curve. Understanding the learning curve for PED is essential for estimating the risk of suboptimal outcomes, guiding decision-making in complex cases, and developing training programs. Although previous literature has shown an association between operator experience and patient outcomes, the learning curve has rarely been visually depicted^9^.

The cumulative summation (CUSUM) analysis is an objective assessment comparing outcomes of consecutive performances against an established standard that has long been used in manufacturing industries for quality control^10^. It has increasingly been applied longitudinally to monitor the outcome and surgical performance^11–13^. In case-control studies, the CUSUM analysis has successfully assessed the learning curve of specific interventions using historical outcome data as a baseline for comparison^14^.

To date, CUSUM analysis has not been applied in neurosurgery. Therefore, the purpose of this study is to evaluate the learning curve for PED treatment and identify the factors that influence procedural safety and success. In this study, the surgical techniques refined during the initial experience gained in PED implantation within the post-market multicenter retrospective research on embolization of intracranial aneurysms with PED in China (PLUS) trial were reported. The learning curve in PED implantation was further analyzed in relation to procedure-related complications and poor functional outcomes in the study cohort. The reproducibility of the learning curve was also evaluated.

## Material and methods

This study has been reported in accordance with the STROCSS (Strengthening the Reporting of Cohort Studies in Surgery) criteria^15^.

### Ethical approval

The study was approved by the Ethics Committee of local institution and conducted in accordance with the principles of the Declaration of Helsinki. Written informed consent was obtained from all study participants prior to enrolment.

### Data source and study design

The inclusion criteria of PLUS trial have been previously reported^4^. Four centres were selected for this study; each having performed more than 20 PED implantations without prior experience with the device. The assistants involved in the procedures also had no prior experience with PED implantation. At each centre, the procedures were carried out by the same neurointerventional surgeon experienced in surgeries such as stent implantation, aneurysm embolization, and vascular malformation embolization. To compare the learning curves for PED, the surgical procedures were categorized as follows: group 1 (first 10 procedures of each centre), group 2 (procedures 11–20), and group 3 (more than 20 procedures).

### Procedural details

The decision to use PED was made by the operators at each study centre, with indications for PED therapy generally including aneurysms that are at risk of failure or recurrence with conventional endovascular techniques (such as large, giant, and wide-necked aneurysms), recurrent aneurysms, fusiform aneurysms, and dissecting aneurysms. At each centre, surgeons followed a similar protocol and guidelines for placing the PED. The procedure was performed with the patient under general anaesthesia and continuous monitoring. An 8F femoral sheath was used as the standard approach for endovascular cases, and an 8F-guiding catheter was selectively placed in the corresponding common carotid artery or subclavian artery. A distal flexible catheter, Navien 5 or 6F (Covidien Neurovascular), was then selectively placed in the internal carotid artery at the level of the petrous segment or distal cervical portion. Next, a Marksman microcatheter (Covidien) was navigated distal to the intracranial aneurysms at the level of the middle cerebral artery through a 14 μm guidewire. The stent tip was deployed in the M1 segment of the middle cerebral artery. For posterior circulation aneurysms, the distal flexible catheter was placed selectively in the vertebral arteries at the level of V2 to V4 segment, and the Marksman microcatheter was navigated to the posterior cerebral artery. During the deployed and expanded of the PED, the tip was positioned in a suitable position to ensure complete coverage of the aneurysm neck and as few branch vessels as possible. Finally, the micro-guidewire was routinely massaged in-stent to increase the degree of stent adherence. Depending on the operator’s preference and experience, additional techniques, such as adjunctive coils, overlapping PEDs, and balloon angioplasty, were also employed.

A combination of aspirin (100–300 mg daily) and clopidogrel (75 mg daily) was the most common dual-antiplatelet regimen. In cases where patients were identified as clopidogrel non-responders, they were given aspirin (100 mg daily) and ticagrelor (90 mg twice daily). The duration of dual-antiplatelet therapy differed from three to over 6 months at each centre, and the dose of aspirin/clopidogrel was adjusted preoperatively after platelet function testing. Platelet function testing was performed in the same manner at all sites.

### Study variables

This study analyzed several variables, including demographic variables, aneurysm variables, procedural variables, major complications, and functional results. Patient data were collected at each study centre during hospitalization and discharge. Procedural success was defined as the successful release of the PED at the targeted landing zone with or without technical adjustments, and the use of additional techniques was also reported. Aneurysm occlusion rates were evaluated through intraoperative angiography. Major complications, including intra/postoperative haemorrhagic events (primarily subarachnoid haemorrhage attributed to aneurysm rupture or distal intraparenchymal haemorrhage), postoperative ischaemic events (transient ischaemic attack and ischaemic strokes), and mass effect deterioration were documented^16^. Other procedure-related complications, such as intraoperative thrombosis and vessel dissection formation, were also considered. The functional outcome was assessed by the modified Rankin Scale score at discharge.

### CUSUM analysis

The CUSUM analysis was conducted to assess the correlation between the learning curve and major complications and functional results. The CUSUM analysis was calculated using the following equation^17,18^:


$$E_{n}\;=\;E_{\left(n-1\right)}+X_{n}\mathrm{with}E_{0}=0,$$

where, $E_{n}$ is the CUSUM after n attempts. $X_{n}$ denotes the result following the nth procedure, with $X_{n}=1-X_{0}$ if a failure occurred and $X_{n}=0-X_{0}$ , if it did not. $X_{0}$ is the established risk or failure rate of the control to which the ongoing attempts are compared. $X_{0}$ can be calculated either as an overall frequency if this is known in this case, or on a case-by-case basis as with paired control trials. In the present study, $X_{0}$ was using 0.15 for major complications and 0.05 for poor outcomes based on the accuracy data provided by Naggara *et al.* ^19^. When $E_{n}$ is plotted for subsequent attempts the gradient of the graph provides information to identify changes in the failure rate: the graph moves upward if the failure rate increases and downward if it decreases. Thus, a change in the gradient from downward to upward following the application of a new intervention serves as an early warning of worse outcomes, even though this may not yet have reached statistical significance.

### Statistical analysis

Continuous variables were presented as mean with SD. Categorical variables were presented as percentages. The baseline characteristics of patients in different surgical groups were compared using the Kruskal–Wallis test for continuous variables and the χ^2^ test for categorical variables. Linear trends of different surgical groups were also tested using one-way ANOVA for continuous variables and the Mantel–Haenszel χ^2^ test for categorical variables.

Multivariable logistic regression models were fitted to evaluate the effect of operator experience on the probability of major complications and poor outcomes (modified Rankin Scale >2) at discharge, adjusting for age, sex, operator, aneurysm size, and location. Odds ratios with their 95% CIs were estimated for different surgical groups.

Given the hypothesis that the effect of increasing operator experience on the major complications and poor outcomes is nonlinear due to a learning process, restricted cubic splines with four knots were used to flexibly model and visualize the association of operator experience with major complications and poor outcomes. Age, sex, operator, aneurysm size, and aneurysm location were mutually adjusted in the spline models. A likelihood ratio test was performed to test for potential nonlinearity, comparing the model with only a linear term against the model with linear and cubic spline terms.

An interaction term was used to conduct a sensitivity analysis to test the hypothesis that the impact of operator experience was different for different individual operators. Linear regressions of the CUSUM score were performed to compare the learning curves of the different operators.

Statistical analyses were performed using SPSS Statistics 26.0 (IBM) and R software v.4.1.3, and all tests were two-sided with a significance level set at *P* less than 0.05.

## Results

### Baseline characteristics

A total of 217 patients with 222 intracranial aneurysms treated with PED were included in our study (Table 1). Of these patients, 75.6% (164/217) were female, and the mean age was 56.0 years (with a range of 16–82 years). Common comorbidities among the study population included hypertension (41.5%, 90/217), cerebral infarction (5.1%, 11/217), diabetes (4.1%, 9/217), cardiac disease (2.8%, 6/217), and hyperlipidemia (2.3%, 5/217). The vast majority of aneurysms were unruptured (96.8%, 210/217). Aneurysm sizes were classified as small (<5 mm) for 11.3% (25/222), medium (5–15 mm) for 45.9% (102/222), large (15–25 mm) for 30.6% (68/222), and giant (>25 mm) for 12.2% (27/222) of the cases. The majority of aneurysms had a saccular morphology (92.3%, 205/222), while 7.7% (17/222) were fusiform. Dissecting aneurysms and partial thrombosis of the aneurysm sac were present in 8.6% (19/222) and 1.0% (2/222) of cases, respectively. The aneurysms were predominantly located in the anterior circulation, with internal carotid aneurysms accounting for 85.1% (189/222) of all cases. Baseline demographics between different surgical groups appeared similar, except for a decreasing trend in aneurysm size in the surgical group with more operator experience.

**TABLE 1:** Comparison of baseline, procedural variables, and complications among patient groups.

Characteristics	Total	Group 1	Group 2	Group 3	*P*	*P* for
	(*n*=217)	1–10 (*n*=40)	11–20 (*n*=40)	>20 (*n*=137)	Value	trend
Demographic variables						
Aneurysms treated with the PED	222	41	42	139	0.780	0.773
Patients with multiple aneurysms, *n* (%)	52 (24.0)	8 (20.0)	7 (17.5)	37 (27.0)	0.376	0.245
Age, years	56.0±11.2	55.3±9.69	56.3±10.38	56.1±11.89	0.780	
Female sex, *n* (%)	164 (75.6)	33 (82.5)	32 (80.0)	99 (72.3)	0.320	0.308
Alcohol abuse, *n* (%)					0.633	0.508
Never	194 (89.4)	36 (90.0)	36 (90.0)	122 (89.1)		
Previous	4 (1.8)	0	0	4 (2.9)		
Current	19 (8.8)	4 (10.0)	4 (10.0)	11 (8.0)		
Smoking, *n* (%)					0.600	0.883
Never	182 (83.9)	34 (85.0)	33 (82.5)	115 (83.9)		
Previous	28 (12.9)	5 (12.5)	7 (17.5)	16 (11.7)		
Current	7 (3.2)	1 (2.5)	0 (0.0)	6 (4.4)		
Hypertension	90 (41.5)	18 (45.0)	18 (45.0)	54 (39.4)	0.732	0.459
Diabetes	9 (4.1)	3 (7.5)	2 (5.0)	4 (2.9)	0.422	0.191
Hyperlipidemia	5 (2.3)	0	0	5 (3.6)	0.224	0.097
Cerebral infarction	6 (2.8)	1 (2.5)	1 (2.5)	4 (2.9)	0.984	0.983
Cardiac disease	11 (5.1)	0 (0.0)	1 (2.5)	10 (7.3)	0.129	0.046
Presentation, *n* (%)					0.275	0.107
Incidental	49 (22.6)	10 (25.0)	13 (32.5)	26 (19.0)		
Symptomatic	161 (74.2)	30 (75.0)	26 (65.0)	105 (76.6)		
Current SAH	7 (3.2)	0	1 (2.5)	6 (4.4)		
PrePED mRS score					0.411	0.220
0–2	214 (98.6)	40 (100.0)	40 (100.0)	134 (97.8)		
3–6	3 (1.4)	0	0	3 (2.2)		
Aneurysms variables						
Aneurysm size, mm*	14.3 ± 7.7	17.8 ± 7.8	13.8 ± 7.0	13.5 ± 7.7	0.007	0.004
Aneurysm location, *n* (%)*					0.078	0.011
Internal carotid artery	189 (85.1)	40 (97.6)	38 (90.5)	111 (79.9)		
Anterior cerebral artery	4 (1.8)	0	0	4 (2.9)		
Middle cerebral artery	9 (4.1)	0	0	9 (6.4)		
Posterior circulation	20 (9.0)	1 (2.4)	4 (9.5)	15 (10.8)		
Saccular form*	205 (92.3)	39 (95.1)	36 (85.7)	130 (93.5)	0.166	0.847
Procedural variables, *n* (%)						
Multiple PEDs used	12 (5.5)	3 (7.5)	2 (5.0)	7 (5.1)	0.833	0.607
Adjunctive coil	136 (62.7)	25 (62.5)	25 (62.5)	86 (62.8)	0.999	0.970
Balloon angioplasty	15 (6.9)	3 (7.5)	3 (7.5)	9 (6.6)	0.967	0.810
Occlusion > 90%	73 (33.7)	14 (35.0)	12 (30.0)	47 (34.3)	0.680	0.731
Complications, *n* (%)						
Major complications	11 (5.1)	4 (10.0)	3 (7.5)	4 (2.9)	0.148	0.053
Haemorrhagic events	6 (2.80)	2 (5.0)	2 (5.0)	2 (1.5)	0.308	0.158
Ischaemic events	4 (1.8)	2 (5.0)	2 (5.0)	0	0.031	0.015
Mass effect	2 (0.9)	0	0	2 (1.5)	0.555	0.318
Vascular dissection	2 (0.9)	0	0	2 (1.5)	0.555	0.318
Intraoperative thrombosis	1 (0.5)	0	0	1 (0.7)	0.746	0.481
Functional results, *n* (%)						
Discharge mRS score					0.043	0.015
0–2	212 (97.7)	37 (92.5)	39 (97.5)	136 (99.3)		
3–6	5 (2.3)	3 (7.5)	1 (2.5)	1 (0.7)		

mRS, modified Rankin Scale; PED, pipeline embolization device; SAH, Subarachnoid hemorrhage.

### Procedural variables

In the study, a total of 230 PEDs were used to successfully treat 222 aneurysms. Adjunctive coils were used in 62.7% (136/217) of patients, and balloon angioplasty was required in 6.9% (15/217) of cases due to insufficient PED expansion. Intraoperative angiography revealed a complete occlusion rate of 33.7% (73/217) immediately following the procedure. There were no significant differences in procedural variables among the surgical groups, although it is worth noting that the proportion of balloon angioplasty was lower in the group with more operator experience, suggesting greater proficiency in device deployment and a reduced need for additional techniques.

### Complications and functional outcomes

**Figure 1:**
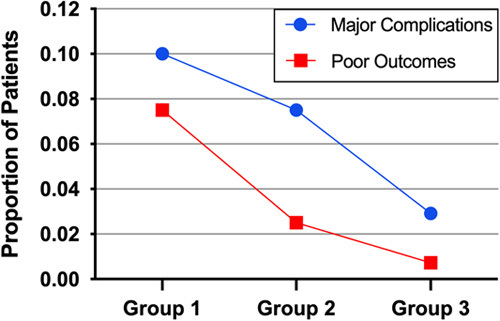
The proportion of major complications and poor outcomes among different patient groups.

The present study observed a low rate of symptomatic procedure-related complications and poor outcomes was observed in the cohort. Specifically, 11 patients (5.1%, 11/217) experienced complications, which included distal intraparenchymal haemorrhage (*n*=1), subarachnoid haemorrhage (*n*=5), ischaemic strokes (*n*=3), transient ischaemic attack (*n*=1), and mass effect deterioration (*n*=1). One patient simultaneously suffered a subarachnoid haemorrhage and ischaemic stroke. Two patients (1.0%, 2/217) experienced procedure-related vascular dissection and one patient (0.5%, 1/217) experienced intraoperative thrombosis. Fortunately, appropriate intraoperative remedy prevented major complications from occurring in these cases. Five (2.3%, 5/217) patients had poor neurological functional outcomes at discharge. Importantly, the rate of major complications decreased from 10.0% (4/40) in group 1 to 7.5% (3/40) in group 2 and only 2.9% (4/137) in group 3 (Figure. 1). Postoperative ischaemic events showed a decreasing trend in group 3 compared with groups 1 and 2 (*P*=0.031 and *P* for trend=0.015), while no significant difference was observed in haemorrhagic events and mass effect deterioration. Similarly, the rate of poor outcomes remarkably fell from 7.5% (3/40) in group 1 to 2.5% (1/40) in group 2 and 0.7% (1/137) in group 3 (Figure.1). Logistic regression analysis indicated a positive association between increased operator experience and decreased risk of poor functional outcomes at discharge (*P*=0.043 and *P* for trend=0.015).

**Figure 2:**
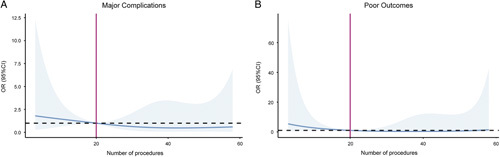
Association of the number of procedures with major complications (A) and poor outcomes (B) in aneurysm patients treated with PED. The estimate is adjusted for case mix accounting for age, sex, aneurysm size, aneurysm location, and operator. P for nonlinearity >0.05. OR, odds ratio; PED, pipeline embolization device.

The relationship between the operator experience and major complications and poor outcomes was linear (Figure.2). Therefore, we adjusted for related risk factors of complications and poor outcomes, including age, sex, aneurysm location, aneurysm size, and different individual operators (Table 2). Although the association with fewer major complications did not reach statistical significance, group 3, with increased operator experience, was still associated with a decreased risk of poor functional outcomes at discharge (odds ratio=0.064, 95% CI=0.005–0.813, *P*=0.034, *P* for trend=0.03). Our findings suggest that increased operator experience may lead to better outcomes in patients treated with PED for intracranial aneurysms.

**TABLE 2:** Adjusted odds ratios of outcomes according to consecutive series of procedures.

Outcomes	Surgical experience	*N*	Events, *n* (%)	Adjusted OR (95% CI)^a^	*P*
Major complications	Group 1	40	4 (10.0)	Ref	Ref
	Group 2	40	3 (7.5)	0.755 (0.150–3.797)	0.734
	Group 3	137	4 (2.9)	0.256 (0.057–1.158)	0.077
*P* for trend				0.063	
Discharge mRS score >2	Group 1	40	3 (7.5)	Ref	Ref
	Group 2	40	1 (2.5)	0.263 (0.024–2.924)	0.277
	Group 3	137	1 (0.7)	0.064 (0.005–0.813)	0.034
*P* for trend				0.030	

mRS, modified Rankin Scale; OR, odds ratio.

a Adjusted for age, sex, operator, giant aneurysm, and posterior circulation aneurysm.

### CUSUM analysis and learning curve assessment

**Figure 3:**
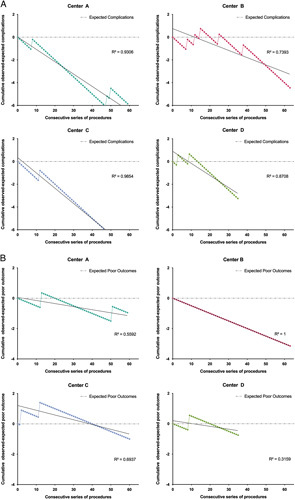
CUSUM analysis for major complications (A) and poor outcomes (B). A downward gradient implies that the intervention (PED) is safe and does not increase risk compared with the control (other endovascular treatment modalities). Any change to an upward gradient should alert the investigators to an increase in the potential risk of the operation (as can be seen between procedures 2–12 of centre C in B). The end of the learning curve is regarded as the point where the failure rate remains at the expected level or lower. The major complications and poor outcomes rate reduced below the expected required up to 27 (mean=13) and 40 (mean=20) cases, varying among the four centres. Dots represent the actual CUSUM score; lines represent the fitted linear regression model. PED, pipeline embolization device.

The CUSUM analyses for major complications and poor outcomes are presented in Figure 3. The plots for major complications and poor outcomes show a downward trend with a high correlation (mean R2=0.88 and 0.64, respectively), indicating that the procedure becomes safer as the operator gains more experience. The end of the learning curve is regarded as the point where the failure rate remains at the expected level or lower. Overall, the rate of major complications and poor outcomes reduced below the expected required up to 27 (mean=13) and 40 (mean=20) cases, varying among the four centres. The operators from four centres performed the PED implantation regularly, resulting in overall low major complications, poor outcomes rates, and stable performance over time.

### Reproducibility of the learning curve

Finally, no interaction between the surgical group and the individual operator was recorded (*P*>0.05). In the linear regression model, the rates of major complications and poor outcomes were found to be comparable (Figure. 3). The slopes for major complications were similar, as were those for poor outcomes.

## Discussion

This study reported on the safety of PED treatment based on the experience of a large series of intracranial aneurysms treated at multiple institutions in China. A CUSUM analysis was employed to evaluate the learning curve for the PED procedure, which had not been previously investigated. It was found that a minimum of 40 cases is required to achieve reproducibility in both complications and functional results.

Clinical medicine still involves much guesswork, with consequences that may be fraught with drama and disappointment for patients and practitioners alike^20^. Thus, doctors prefer to evaluate disease progression and treatment efficacy whenever possible. CUSUM analysis is a straightforward statistical method that has been used in several medical fields to evaluate operator learning curves and as a continuous quality assurance indicator. The primary application of CUSUM analysis is to assess operator learning curves and training for new techniques or procedures. Real-time comparisons can be conducted between control groups or previously established data by collecting prospective outcomes. Moreover, CUSUM analysis has the potential to be used as an early warning detector in clinical trials and a quality assurance indicator for operator revalidation.

The present study demonstrates that PED is associated with low complications and mortality rates for intracranial aneurysms that are often deemed high risk or unsuitable for traditional embolization strategies. The major complications and poor outcomes rates (5.1% and 2.3%, respectively) in our study population are consistent with previously published PED series. A multinational retrospective study of PED involving 17 centres worldwide reported a 7.1% complication rate and 3.8% neurologic mortality in a large cohort of 793 patients^16^. In a pooled analysis of 3 large studies that included 1092 patients with 1221 aneurysms, the major neurological morbidity and mortality were 5.7% and 3.3%^21^.

Subarachnoid haemorrhage and distal intraparenchymal haemorrhage are the most dreaded complications associated with PED. Of the five patients who experienced poor functional outcomes in our study, four reported haemorrhagic complications, and delayed aneurysm rupture may be the primary cause of the other subarachnoid haemorrhages. The potential mechanisms underlying these complications remain uncertain, although several hypotheses have been proposed. Proximal migration or shortening of the PED with the formation of rapid blood flow at the aneurysmal neck directed into the aneurysm sac may be a possible mechanism underlying delayed aneurysm rupture. Aneurysm wall weakening caused by inflammation and proteolytic enzymes during thrombosis may be another potential factor. Also, increased intra-aneurysmal pressure following the PED treatment may lead to rupture^22^. Accordingly, some operators prefer to use adjunctive intraluminal coils to attenuate the effects of jet flow, intra-aneurysmal pressure, and intra-aneurysmal thrombosis. However, the efficacy of this strategy remains unclear, as four of the five patients with delayed aneurysm rupture in this study received adjunctive coils. Conversely, adjunctive coiling was an independent predictor of mass effect deterioration and poor functional outcomes in the entire PLUS cohort^4^.

Perioperative platelet over-inhibition may also put patients at risk of developing haemorrhagic complications. The pre-procedural baseline P2Y12 value was reported to be an independent predictor of perioperative haemorrhagic and thromboembolic complications^23,24^. Although previous studies suggested that ipsilateral distal intraparenchymal haemorrhage may be associated with antiplatelet regimens^23,25^, this complication is not commonly seen in conventional aneurysm coiling. The flow diversion process increases flow shear stress, which activates the platelets exposed to the metal surface area. Subsequently, activated platelet plug embolization distally, ischaemic infarction, and secondary haemorrhagic transformation may occur^25^. In the present study, the only patient with ipsilateral distal intraparenchymal haemorrhage also suffered a perioperative ischaemic stroke, which further strengthens this hypothesis. Ischaemic complications were more frequently observed than haemorrhagic complications, which is in contrast to previous studies. Possible mechanisms for ischaemic complications include the microemboli originating from the treated vessel, atherosclerotic plaques scraped off the great vessel wall by catheter manipulation, and procedure-related vascular dissection.

A learning curve represents the rate at which skills or experiences are acquired in a given period from manual procedures. This well-recognized phenomenon is also applied to neurointerventional surgeries, including but not limited to carotid artery stenting and coil embolization of intracranial aneurysms^26,27^. PED embolization represents a fairly challenging procedure requiring better technical flexibility and sophisticated catheter-based skills than standard embolization strategies. Therefore, to acquire minimal technical competence for this intervention, the manufacturer suggests the initial five PED procedures be performed under the supervision of an approved specialist. Our study also observed a definite procedural learning curve for PED treatment consistent with previously reported^9^. The rate of major complications declined from 7.5–10% in groups 1 and 2 to 2.9% in group 3, and the rate of poor functional outcomes decreased from 7.5% during the first ten procedures to 2.5% after this threshold and only 0.7% in the third group. It should be noted that all intraoperative accidents, including two procedure-related vascular dissections and one intraoperative thrombosis, occurred in group 3. However, the number of patients in group 3 was much larger than in groups 1 and 2, and the increased operator experience may have prevented these accidents from becoming major complications. Jabbour *et al.* ^9^ mentioned that the decreasing complication rate could be secondary to the evolution of the PED deployment technique (devices per aneurysm from 1.6 to 1.2). We also observed decreasing major complications and poor outcomes rates even if there was no difference in the amount of PEDs used between surgical groups. These findings suggest the presence of an initial learning curve for PED treatment and that increased operator experience is associated with procedural safety.

Our findings support the linear relationships between operator experience and major complications and poor outcomes. Although there is no clear distinction between a plateau phase and a learning phase, the risk of major complications and poor outcomes dropped rapidly over the initial 20 procedures. Notably, this study represents the first description of the learning curve for PED treatment based on CUSUM analysis. The learning curve for major complications decreased below the expected line after the average procedure 13 and for poor outcomes after an average of 20 procedures. Therefore, up to 20 formal training procedures may be necessary to reduce complications, and extensive experience is needed to achieve a very low risk of poor outcomes. It is essential to consider the learning curve when considering PED treatment. It is worth noting that while billions are invested in bio-information research at all levels, little effort is dedicated to understanding how surgeons improve their performance, which could have significant benefits in achieving better patient outcomes.

In addition to the learning curve for the individual operator, institutional experience also plays a crucial role in the success of new procedures. In our cohort, the interventional risk for PED rookie interventionalists performing PED treatment remained relatively low due to the presence of an experienced team that managed incipient complications. Thus, safety was somewhat assured at experienced centres, even if an individual operator had not yet fully acquired the technical competence for the procedure. However, it remains to be seen whether low-volume centres can achieve similar technical success in increasing the safety of new procedures as high-volume centres. Further studies are needed to address this issue.

This study is not devoid of limitations. First, it is limited by its retrospective design. Additionally, the study population consisted mainly of patients with complex aneurysms, and the procedures were performed by skilled neurointerventional surgeons. Furthermore, this multicenter study did not analyze any infrastructure variables that could have influenced the outcomes. However, we ensured that all centres had similar levels of resources and equipment. Lastly, the absence of the evaluation of the learning curve analyzing the timing though the relationship between timing and experience is open to some debate. Future studies could investigate whether low-volume centres are able to achieve the same level of technical success and safety as high-volume centres in the treatment of PED complications. It may also be worthwhile to explore whether certain factors, such as patient selection and case complexity, influence the learning curve for PED treatment.

## Conclusions

Our study demonstrated that CUSUM analysis is a reliable tool for continuous monitoring of surgical performance. Specifically, our results showed that performing 40 cases of PED treatment is necessary to achieve reproducibility in terms of both complications and functional outcomes. Furthermore, the rate of major complications and poor outcomes decreases significantly after the first 20 procedures. These findings highlight the importance of adequate training and experience for operators performing PED treatment, and support the use of CUSUM analysis as a valuable tool for quality control in neurointerventional procedures.

## Ethical approval

The study protocol was reviewed and approved by the ethics committee of Beijing Tiantan Hospital, and the approval number given by the ethical board was KY 2018-098-02.

## Sources of funding

This study was sponsored by the National Natural Science Foundation of China (82071302) and Bai Qian Wan Talent Plan (2017A07).

## Author contribution

Ju.L. and Ya.Z. conceived the idea, designed the paper, and wrote the manuscript. Ju.L. performed the statistical analysis. Ya.Z. collected the data. H.Z., T.L., D.S., S.G., A.M., Yu.W., W.F., Ya.W., J.W., G.M., H.S., X.Y., Ji.L., and Yu.Z. funded the study, critically revised the manuscript and approved the final manuscript as submitted. Yu.Z. is the guarantor. All authors agreed to be accountable for all aspects of the work in ensuring that questions related to the accuracy or integrity of any part of the work are appropriately investigated and resolved.

## Conflicts of interest disclosure

The authors declare no conflicts of interest.

## Research Registration Unique Identifying Number (UIN)

1. Name of the registry: Post Market Multi-Center Retrospective Research on Embolization of Intracranial Aneurysms With Pipeline Embolization Device in China.

2. Unique Identifying number or registration ID: NCT03831672.

3. Hyperlink to your specific registration (must be publicly accessible and willbe checked): https://beta.clinicaltrials.gov/study/NCT03831672.

## Guarantor

Junlin Lu, Yang Zhao.

## Data statement

The data that support the findings of this study are available from the corresponding author upon reasonable request.

## Provenance and peer review

Not commissioned, externally peer-reviewed.

## Acknowledgements

The authors thank the patients and radiologists who participated in this study for their help.
